# Effects of a 9-weeks arch support intervention on foot morphology in young soccer players: a crossover study

**DOI:** 10.1186/s13102-022-00590-3

**Published:** 2022-11-14

**Authors:** Kohei Hikawa, Toshiharu Tsutsui, Takehiro Ueyama, Jin Yang, Yukina Hara, Suguru Torii

**Affiliations:** 1 Graduate School of Sport Sciences, Waseda University, Tokorozawa, Saitama Japan; 2 Faculty of Sport Sciences, Waseda University, 2-579-15 Mikajima, Tokorozawa, Saitama 359-1192 Japan

**Keywords:** Flat foot, Arch support, Intrinsic foot muscles, Extrinsic foot muscles

## Abstract

**Background:**

A flat foot is a common cause of chronic sports injuries and therefore many opportunities for arch support interventions exist. However, young athletes change their foot morphology due to developmental influences even without intervention. Therefore, developmental influences need to be considered when examining the effects of arch support, but there have not been sufficient longitudinal studies to date. This study aimed to determine the effect of the arch support intervention by performing a 9-weeks arch support intervention on the foot morphology and cross-sectional area of the foot muscles in flat-footed young athletes. Thirty-one elementary school boys (Age 11.4 ± 0.5 years, Height 145.2 ± 7.4 cm, Weight 38.8 ± 8.3 kg, BMI 18.2 ± 2.2 kg/m^2^) with a decreased medial longitudinal arch in the foot posture index were selected as participants from a local soccer club and randomly divided into two groups.

**Methods:**

In one group, in the intervention period, an existing arch supporter was used to provide arch support, while in the other group, no special intervention was provided in the observation period. To account for developmental effects, the intervention study was conducted as an 18-weeks crossover study in which the intervention and observational phases were switched at 9 weeks after the intervention. Foot morphology was assessed using a three-dimensional foot measuring machine, and the cross-sectional area (CSA) of the internal and external muscles of the foot was assessed using an ultrasound imaging device. We examined the effect of the intervention by comparing the amount of change in the measurement results between the intervention and observation periods using corresponding t-tests and Wilcoxon signed-rank sum test, analysis of covariance methods.

**Results:**

After adapting the exclusion criteria, 14 patients (28 feet) were included in the final analysis. The CSA of the abductor hallucis muscle (ABH) increased 9.7% during the intervention period and 3.0% during the observation period (p = 0.01). The CSA of the flexor digitorum longus muscle (FDL) increased 7.7% during the intervention period and 4.2% during the observation period (p = 0.02).

**Conclusion:**

A 9-weeks arch supporter intervention may promote the development of the ABH and FDL CSA in young flat-footed soccer players.

## Background

A flat foot is a chronic foot condition associated with a reduction in the medial longitudinal arch (MLA) of the foot, rearfoot medial arch, and midfoot abduction to the rearfoot side [1]. The MLA is critical for shock absorption and propulsion during movement [2], and its dysfunction can result in chronic sports disorders such as medial tibial stress syndrome and plantar fasciitis [3, 4]. Flat foot rehabilitation is an important treatment option. The bodies of young athletes are still developing, and their tissues are more fragile than those of adults. In addition, during this period, the increase in height and weight has an effect, and sports injuries of the feet and lower limbs increase [5]. Interventions for flat feet are therefore particularly important to reduce mechanical stress.

Flat foot rehabilitation involves the use of insoles and other devices ("arch support") to support the MLA and promote its formation. Arch support has been reported to have (1) kinematic and kinetic effects such as a decrease in the angle of rear foot abduction [6] and tibial internal rotation [7] during running, (2) shock absorption and load distribution effects such as a decrease in vertical impact force [8] and pain relief by load distribution [9] during walking and stair climbing, and (3) changes in electromyographic amplitude of the tibialis posterior and peroneus longus muscles [10] during walking. Multiple studies have revealed these effects.

Although the use of arch support has been investigated from various perspectives, these are mostly cross-sectional studies that have analyzed only the immediate effects. In a longitudinal study, Jung [11] reported an increase in the cross-sectional area (CSA) of the abductor hallucis (ABH) muscle, which is associated with MLA dynamic stability, after an 8-weeks arch support intervention during outdoor walking in adult flat-footed participants. Thus, longitudinal studies have also suggested that arch support is useful to support the MLA. Although a number of clinical interventions are performed during the growth period, longitudinal studies are rare [12]. Foot morphology changes are higher during the growth period due to developmental influences [13]. Therefore, it is critical to investigate the longitudinal effects of the arch support. In addition, based on past reports, it is not clear whether the changes during arch support interventions for young athletes, which are often performed in clinical practice, are due to developmental influences or arch support. A previous study reported that arch support for adults causes atrophy of the intrinsic foot muscles [14], and it is possible that arch support for young athletes may inhibit foot development. Therefore, the effectiveness of arch supports for young flat-footed athletes should be determined based on developmental influences through longitudinal studies, and their clinical use should be based on a thorough understanding of their effectiveness. Practically, if the results of this study are positive, it provides a rationale for the use of arch supports for the purpose of promoting foot development, such as for the prevention of flat foot. On the other hand, arch supports have also been reported to contribute to the development of foot muscles in adults [11]. They have also been reported to promote the development of the MLA morphology during systemic development [12]. Therefore, we believe that arch support intervention in young athletes has a positive impact on promoting the development of foot muscles and the MLA [14].

The purpose of this study was to determine the effect of the arch support intervention by performing a 9-weeks arch support intervention on the foot morphology and cross-sectional area of the foot muscles in flat-footed young athletes.

## Method

All methods were implemented according to relevant guidelines and regulations.

### Participants

**Fig. 1:**
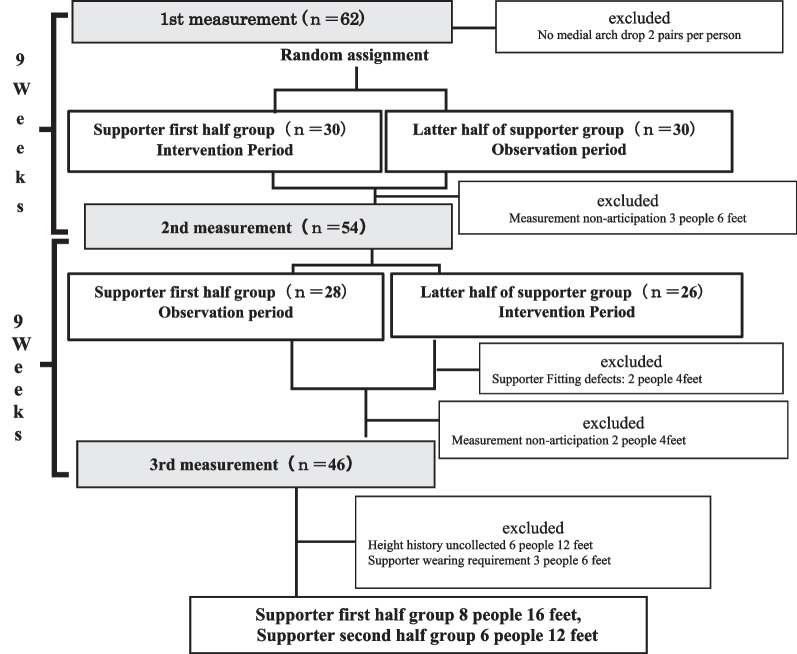
Flowchart of participants through the study period. A 9-weeks intervention period and a 9-weeks observation period for a total of 18 weeks of crossover study and 3 times measurement

We distributed a recruitment form to recruit elementary school boys who can volunteer from a soccer club near the facility to which the author belongs. Prior to measurements, the purpose of this study was explained in writing and orally to the participants and their guardians, and written consent was obtained. This study was conducted after receiving approval (approval number: 2020-242) from the “Ethics Review Committee for Research Involving Human Subjects” of Waseda University. The exclusion criteria were as follows: those who did not agree with the purpose of the study, those who had pain in the lower limbs, those who could not participate in all three measurements, those with missing data, those who were unable to wear the arch support for an average of at least 1 h per day during the intervention period, and those with no decline in the MLA were excluded (Fig. 1). The sample size was estimated by G*Power 3.1.9.2 software. The minimum sample size was calculated to be 21, considering the previous studies [11, 14], and based on an effect size of 0.50, an α-level of 0.05, and a power of 0.80. Finally, considering possible dropouts due to COVID-19and long-term intervention, 31 participants were recruited.

### Outcome measurement

#### Classification of foot morphology

The foot posture index-6 (FPI) was used to classify foot morphology [1]. The FPI is an evaluation tool developed considering its simplicity and convenience. FPI scores of + 1 and + 2 feet in the MLA shape item were defined as flat feet with low MLA and were included in the intervention. A physical therapist with 7 years of clinical experience who has a history of clinical examinations and graduate studies research conducts FPI measurements alone after confirming in advance that sufficient reproducibility can be obtained.

#### Foot morphology evaluation

**Fig. 2:**
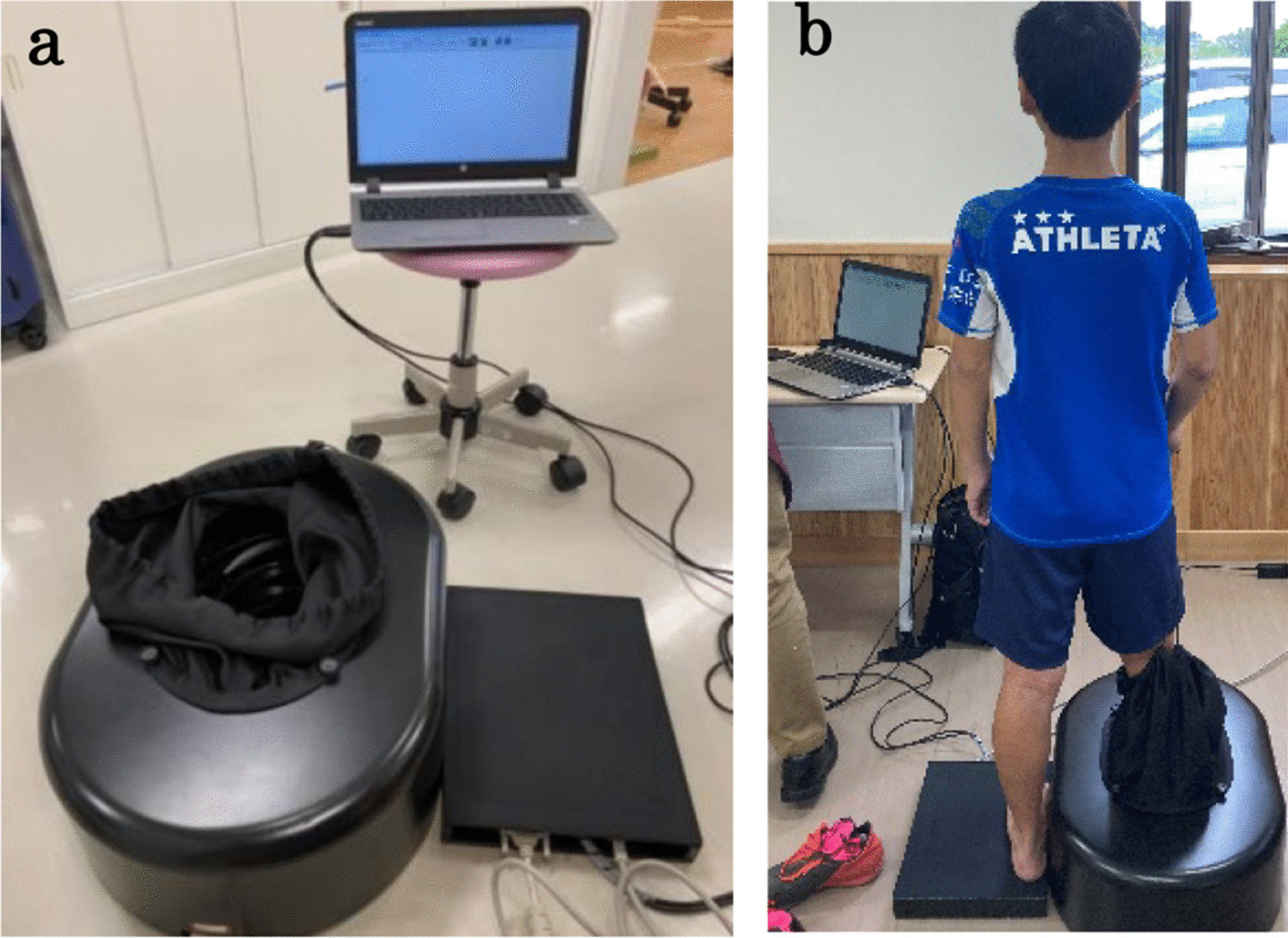
3D foot measuring machine. **a** Measuring equipment. **b** Measuring scene

An automatic three-dimensional foot measuring machine (Real Foot, Dream GP Inc, Osaka, Japan) was used to evaluate foot morphology (Fig. 2). Measurements were taken in static standing (on both feet) and sitting positions after marking the scaphoid bone length. The measurements included foot length (from the back of the calcaneus to the tip of the longest toe), foot width (from the first metatarsal to the fifth metatarsal head), foot circumferences (circumference at the first metatarsal head—fifth metatarsal head), and navicular height (from the floor surface to the lowest end of the navicular rough surface). The difference in navicular height between the upright and seated positions, the navicular drop, was expressed as an index of arch morphology and also as arch height ratio (Standing navicular height divided by foot length times 100). All measurements were performed at the same place and at approximately the same time. Measurements were performed by a single, well-practiced and appropriate technique person.

#### Muscle cross-sectional area evaluation of intrinsic and extrinsic foot muscles

**Fig. 3:**
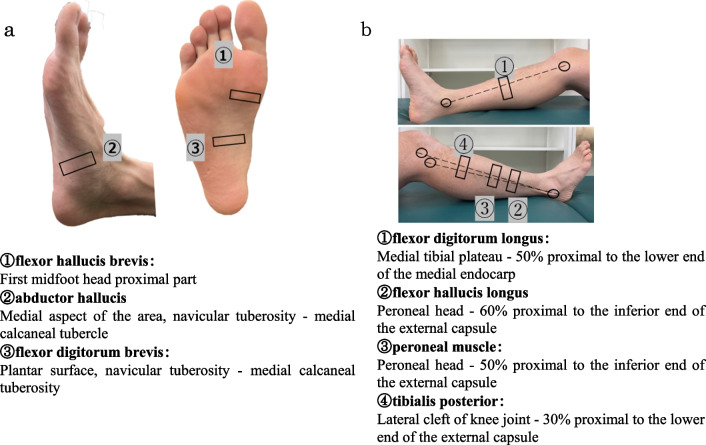
Measurement of muscle cross-sectional area by ultrasound imaging system. **a** Measurement site of intrinsic foot muscles. **b** Measurement site of the extrinsic foot muscles

The intrinsic and extrinsic foot muscles were measured using an ultrasound imaging system (SonoSite Edge II, FUJIFILM SonoSite, Inc, USA) in the B-mode. A probe (Linear Probe HFL38xp, FUJIFILM SonoSite, Inc, USA) with a frequency of 6–13 MHz was used for the measurements. This is approximately the same frequency used in previous studies [14–16]. In addition, at this frequency, it was used after confirming in advance that the muscle of the measurement site could be imaged in a sufficient state for analysis with several hundred legs. The intrinsic muscles of the foot flexor hallucis brevis (FHB) muscle, abductor hallucis (ABH) muscle, and flexor digitorum brevis (FDB) muscle were placed in a dorsal recumbent position with the knee in slight flexion and the ankle in slight plantar flexion. The extrinsic ankle muscles—the flexor digitorum longus (FDL) muscle, flexor hallucis longus (FHL) muscle, peroneal (PER) muscle, and tibialis posterior (TP)—were placed in an end-sitting position with the ankle joint in the mid-position, the knee joint in 90° flexion, and the hip joint in 90° flexion. The participants were instructed to relax their feet without applying pressure to the lower leg, and then the measurements were performed. The FHB and FDB muscles were measured by applying a probe in the short-axis direction to the proximal portion of the first metatarsal head [15], ABH and FDB to the medial aspect [14] and plantar surface [14] of the foot between the navicular tuberosity and medial tubercle of the calcaneus, respectively (Fig. 3a). The FDL, FHL, PER, and TP were imaged by applying a short-axis probe to the proximal 50% [16] of the medial end of the tibial plateau and the inferior end of the medial end of the medial tibial plateau, the 60% [17] of the proximal end of the fibular head and the inferior end of the external capsule, the 50% [16] of the proximal end of the fibular head and the inferior end of the external capsule, and the 30% [18] of the proximal end of the lateral knee joint cleft and the inferior end of the external capsule, respectively (Fig. 3b). A physiotherapist with 7 years of clinical experience who has experienced clinical examinations and graduate studies confirmed the high reliability of the measured values in preliminary experiments, and performed all measurements and data analysis by himself.

#### Assessment of developmental age

Since the level of foot growth differs depending on the biological maturity [13], the peak height velocity age (PHVA), an index of biological maturity, was calculated using the BTT method and examined. The BTT method estimates physical maturity by assessing predicted height structure using the Bock, Thyssen and du Toit (BTT) mathematical structural growth model in AUXAL software [19]. PHVA, the age at which height increases the most, was estimated from the history of each participant's height data using a dedicated software (AUXAL3.1, Scientific Software International Inc, USA). Developmental age is the difference between age and PHVA at the time of measurement and is an indicator of maturity [20].

#### Procedure

To assess the developmental effects, the intervention study was conducted as an 18-weeks crossover study (Fig. 1). The study protocol included a pre-intervention session prior to the start of the experiment, in which an overview of the experiment, the wearing method of the arch supporter, and precautions were explained. Obtained consent before conducting the experiment. The participants were randomly divided into two groups: the first-half supporter group received a supporter intervention period of 9 weeks in the first half and an observation period of 9 weeks in the second half. The second-half supporter group received an observation period of 9 weeks in the first half and a supporter intervention period of 9 weeks in the second half. The intervention and observational phases were switched at 9 weeks after the intervention. Since a previous report showed that the intervention was effective after 8 weeks in adults [11], the intervention period was 9 weeks. For the allocation, one examiner prepared a list table in the order in which the participants arrived at the measurement site. Another examiner blinded the measurement results and randomly allocated numbers 1 and 2 from the top of the list table, dividing them into two groups. The first group was designated as the first half supporter group and the second group as the second half supporter group. Measurements were taken at three time points: pre-intervention, midterm, and post-intervention. For the results, the difference between the second measurement and the first measurement was defined as the period I change, and the difference between the third measurement and the second measurement was defined as the period II change.

**Fig. 4:**
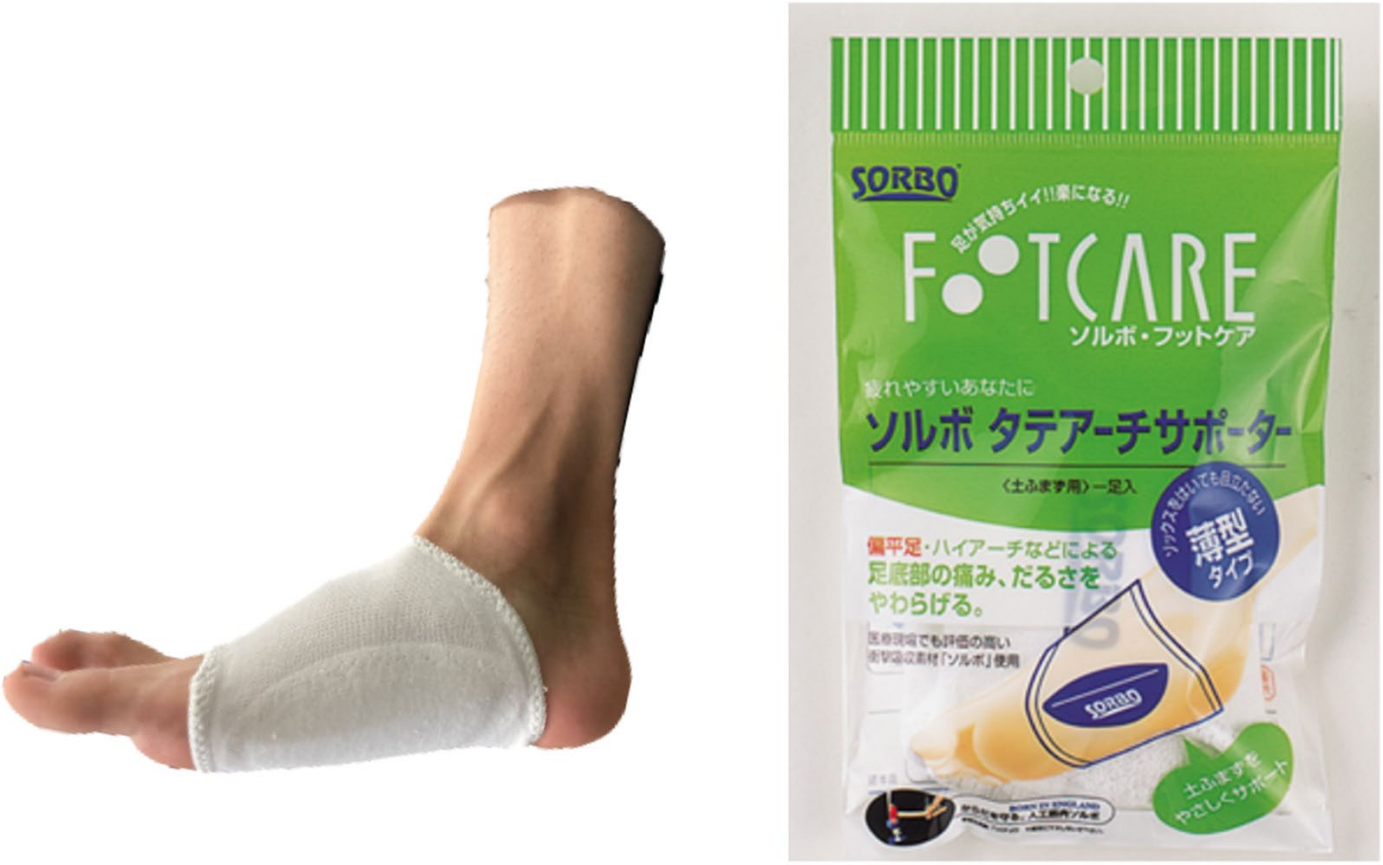
Arch supporter. (Solvo-Tate Arch Supporter, Sanjin Sangyo Co Ltd, Saitama, Japan)

For the arch support, an arch supporter (Solvo-Tate Arch Supporter, Sanjin Sangyo Co Ltd, Saitama, Japan) with an arch pad made of a viscoelastic polymer material attached to a stretchy knit was used (Fig. 4). The small (S) or large (L) size of the supporter was selected according to the participant’s foot length. The height of the arch pad was 8 mm for size S and 10 mm for size L, and the cloth thickness was 1 mm). The method of wearing the supporter was explained using the manufacturer’s instructions, according to which the pads attached to the supporter were aligned with the medial arch of the foot and worn barefoot with socks worn over the top. The participants were instructed to wear shoes for as long as possible, except during strenuous exercise, sleeping, and bathing. They were also instructed to record the time for which the arch supporters were worn. An in-person site visit was conducted between the 4th and 5th weeks of intervention where the examiner evaluated the wearing method of the arch supporter and the wearing time using questionnaires. Additional instructions were provided as needed to increase compliance with the arch supporter use.

#### Statistical analysis

We compared the means of the two groups’ physical characteristics by using either an unpaired t-test or the Mann–Whitney U test. To examine the effect of the intervention, the sum of the means of the change in the first half of the supporter group in period I and the change in the second half of the supporter group in period II and the change in the first half of the supporter group in period II and the change in the second half of the supporter group in period I were compared using a corresponding t-test for items for which normality was found and a t-test for items for which normality was not found. The Wilcoxon signed-rank sum test was used for items that were not identified. For items that were indicated to be significant by the corresponding t-test or Wilcoxon signed-rank sum test, an additional analysis was performed using analysis of covariance (ANCOVA) with the developmental age as a covariate to take into account the effects of growth. The significance level was set at less than < 0.05, and the effect size was calculated using Cohen's d (small: 0.20, medium: 0.50, and large: 0.80). A statistical software (SPSS Statistics 27, IBM, USA) was used for the statistical analysis.

## Results

### Intervention results (basic attribute results)

The experimental group consisted of 31 participants (62 feet). One person with no medial arch reduction (2 feet), 5 people who could not participate in all three measurements (10 feet), 5 people who could not wear the supporter until the end because of pain or non-compliance (10 feet), and 6 people whose developmental age could not be calculated because their past height history was unknown (12 feet) were excluded from the analysis (Fig. 1). After applying the exclusion criteria, the final analysis included 28 feets of 14 people (16 feets of 8 supporters in the first-half supporter group and 12 feet of 6 supporters in the second-half supported group). Wearing compliance was 80% after adapting to the arch supporter wearing conditions. The participants in the analysis wore the arch supporter for an average of at least 5 h per day during the intervention period and showed adequate compliance (Table 1). The basic attributes of each group at the time of the initial measurement are shown in Table 1. There were no differences between groups in maturity level or time of wearing the supporter other than weight (p = 0.01) and Body mass index (p = 0.01).

**Table 1:** Participant characteristics

	Supporter first half group (n = 16)	Latter half of supporter group (n = 12)	p Value
	Mean (SD)	Mean (SD)	
*Basic properties*			
Age (age)	11.5 (0.5)	11.6 (0.5)	0.56
Developmental age (age)	− 1.09 (1.9)	− 1.23 (0.8)	0.81
Height (cm)	144.6 (7.1)	150.1 (9.8)	0.095
Body weight (kg)	36.7 (5.2)	45.2 (10.5)	0.01*
Body mass index (kg/m^2^)	17.5 (1.4)	19.8 (2.2)	0.01*
Foot length (cm)	22.8 (1.1)	23. 6 (1.6)	0.09
Foot posture index (Point)	6.5 (2.0)	5.4 (2.4)	0.21
Supporter wearing time (hours)	6.5 (2.1)	5.0 (3.3)	0.15
Practice time (hours/week)	4.5	4.5	

*SD* Standard deviation

*Indicates statistical significance at p < 0.05

### The intervention effectiveness of arch support

The CSA of ABH increased 3.0% at 0.06 ± 0.06 cm^2^ during the observation period and 9.7% at 0.17 ± 0.12 cm^2^ during the intervention period, indicating a significant intervention effect (p = 0.01, d = 1.12; Table 2). The CSA of FDL increased by 4.2% at 0.09 ± 0.08 cm^2^ during the observation period and by 7.7% at 0.14 ± 0.08 cm^2^ during the intervention period, indicating a significant intervention effect (p = 0.02, d = 0.61; Table 2). No intervention effects were observed for other foot morphologies or CSA (Table 2).

**Table 2:** Examining intervention effects with corresponding t-tests

	Observation period (n = 28)	Intervention period (n = 28)	Mean difference (95%CI)	p Value	Effect size (d)
	Mean (SD)	Mean (SD)			
*Basic properties*					
Height (cm)	0.013 (0.004)	0.011 (0.07)	0.002 (− 0.001, 0.006)	0.15	0.38
Body weight (kg)	1.36 (1.3)	1.45 (1.1)	− 0.09 (− 0.74, 0.56)	0.79	0.07
Body mass index (kg/m^2^)	0.26 (0.50)	0.36 (0.49)	− 0.11 (− 0.37, 0.16)	0.41	0.22
*Foot morphology*					
Foot length (mm)	− 0.42 (7.4)	2.55 (2.8)	− 2.96 (− 5.99, 0.05)	0.054	0.53
Foot width (mm)	0.07 (1.7)	0.23 (1.7)	− 0.16 (− 1.10, 0.766)	0.73	0.10
Foot circumference (mm)	− 0.15 (4.5)	ｰ0.63 (4.0)	0.47 (− 1.80, 2.75)	0.68	0.11
Standing navicular bone height (mm)	− 0.13 (5.5)	− 0.26 (5.1)	0.14 (− 2.72, 2.99)	0.92	0.03
Arch height rate (%)	− 0.47 (2.8)	0.14 (2.4)	1.63 (− 0.62, 3.87)	0.38	0.24
Navicular drop (mm)	− 0.14 (3.4)	− 1.77 (4.9)	− 0.61 (− 1.99, 0.77)	0.15	0.39
*Foot muscle cross-sectional area*					
Flexor hallucis brevis CSA (cm^2^)	0.04 (0.05)	0.05 (0.06)	− 0.01 (− 0.04, 0.01)	0.42	0.21
Abductor hallucis CSA (cm^2^)	0.056 (0.06)	0.166 (0.12)	− 0.11 (− 0.16, − 0.06)	0.01*	1.12
Flexor digitorum brevis CSA (cm^2^)	0.04 (0.07)	0.06 (0.09)	− 0.02 (− 0.07, 0.02)	0.26	0.31
Flexor digitorum longus CSA (cm^2^)	0.09 (0.08)	0.14 (0.08)	− 0.05 (− 0.09, − 0.01)	0.02*	0.61
Flexor hallucis longus CSA (cm^2^)	0.06 (0.06)	0.09 (0.11)	− 0.03 (− 0.08, 0.01)	0.15	0.40
Peroneus muscles CSA (cm^2^)	0.12 (0.11)	0.11 (0.11)	0.01 (− 0.04, − 0.07)	0.61	0.14
Tibialis posterior muscle CSA (cm^2^)	0.14 (0.11)	0.12 (0.10)	0.01 (− 0.05, 0.07)	0.75	0.09

*SD* Standard deviation, *95%CI* 95% confidence interval, *CSA* Cross sectional area

*Indicates statistical significance at p < 0.05

*Effect size: Cohen's d (small: 0.20 medium: 0.50 large: 0.80)

### Investigating intervention effects with developmental influences

The CSA change in ABH was 0.06 ± 0.1 cm^2^ during the observation period and 0.17 ± 0.1 cm^2^ during the intervention period, with a significantly greater increase during the intervention period (p = 0.01; Table 3). The CSA change in FDL was 0.09 ± 0.1 cm^2^ during the observation period and 0.14 ± 0.1 cm^2^ during the intervention period, with a significantly greater increase during the intervention period (p = 0.03; Table 3).

**Table 3:** Results of ANCOVA analysis of intervention effects with maturity

	Observation period(n = 28)	Intervention period(n = 28)	Mean Difference (95%CI)	p Value
	Mean(SD)	Mean(SD)		
Abductor hallucis CSA (cm^2^)	0.06 (0.1)	0.17 (0.1)	0.11 (0.06,0.16)	0.001*
Flexor digitorum longus CSA (cm^2^)	0.09 (0.1)	0.14 (0.1)	0.05 (0.01,0.09)	0.03*

*SD* Standard deviation, *95%CI* 95% confidence interval, *CSA* Cross sectional area

*Indicates statistical significance at p < 0.05

## Discussion

This is the first intervention study to examine the effect of an approximately 9-weeks arch support intervention on foot morphology in young flat-footed soccer players. This took into account the developmental effects using a crossover study and found that the intervention contributed to an increase in CSA in ABH and FDL muscles.

As hypothesized, the arch support contributed to an increase in the CSA of the foot muscles. ABH is the most medial of the intrinsic foot muscles attached from the calcaneus to the first metatarsal phalanx and the seed bone [21]. It is an intrinsic foot muscle involved in arch function by flexing the toes, medial calcaneal flexion, and ankle joint rotation [22]. The flexor digitorum longus originates from the middle third of the posterior surface of the tibia and attaches to the second and fifth phalanges. The FDL is an extrinsic foot muscle that flexes the toes and, like the ABH, is involved in arch function [23]. Since the participants in this study were those with a reduced MLA, it can be inferred that the muscle fiber lengths of the ABH and FDL were in an elongated position compared to those in individuals with a normal MLA. According to the muscle length-tension curve [24], active muscle tension is reduced when the muscle fibers are in an overstretched position. It can be inferred that the arch support intervention changed the alignment of the MLA, resulting in a change in the length of the muscle fibers in the ABH and FDL to a length that is more favorable for exerting muscle tension compared to the stretched position. Therefore, it can be inferred that the active muscle tension of the ABH and FDL increased and muscle function improved. We believe that the improvement in muscle function stimulated neuromuscular function during ABH and FDL activities in daily life and that the continuous intervention for 9 weeks contributed to the change in CSA. Other reported effects of arch support include changes in muscle activity [25], improvement in balance ability [26], and activation of sensory receptors [27], and it is possible that these effects contributed to the increase in CSA.

Considering the effects of growth, the CSA of the ABH increased by 9.7% during the intervention period and 3.0% during the observation period. The CSA of the FDL increased by 7.7% during the intervention period and 4.2% during the observation period. Jung et al. [11] reported a 5.1% increase in the CSA of the ABH in the arch support group after an 8-weeks outdoor walking intervention in adults with flat feet. We believe that this difference in the intervention effect is due to differences in the studied population. The present study was conducted on young athletes, whereas the study by Jung et al. was conducted on adults. In adults, foot development is complete, but young athletes in the age group of this study are in a period of active foot development [13]. Therefore, we expected that interventions for young athletes would have a higher intervention effect due to the increase in CSA than in adults because physiological development also affects the results in addition to the intervention effect of arch support alone.

Although hypothesized to contribute to the development of foot morphology, the results of this study did not reveal these effects on young athletes. This may be due to the short intervention period of this study. Previous studies on children have reported intervention effects on arch morphology, pain, and movement at 12 weeks or several years [12, 28]. In this study, a medium effect size (d = 0.53) was found for the foot length item, although the t-test results did not show a statistically significant difference. In this study, we believe that an intervention period of 12 weeks or more, rather than 9 weeks, and an increase in the number of participants would have clarified the effect of the intervention on foot morphology. However, since there was no difference in foot morphology development between the intervention and observation periods, the arch supports do not negatively inhibit the development of [14] foot morphology, as in previous studies. Thus, it is considered safe to use the arch support during the growth period. In addition, there was no intervention effect on CSA such as the TP. The reason for this is thought to be that the method of extracting participants has a large effect. This study was conducted to select participants only with decreased MLA in FPI. Therefore, it is possible that some of the selected participants included MLA decline associated with pronation and apparent MLA decline. Given the length-tension curve of the muscle with alignment improvement due to the intervention effect of arch support [4], For muscles that adhesion oblique to the MLA, such as TP [29], it can be inferred that there is variation in the effectiveness of the intervention when arch support intervention is performed. For these reasons, we believe that no significant differences were found in TP and other factors. In the future, we believe that the effect of arch support intervention can be unified by classifying the foot morphology in combination with the evaluation of rear foot alignment and navicular drop.

This is the first study to examine the effects of arch support interventions in young flat- footed athletes. The results of this study demonstrate the importance of foot environment during the growth period. This is a great finding when considering the prevention of flat feet during the developmental period. We believe that the usefulness of arch support for disability prevention can be examined by longitudinally investigating performances, such as walking movements and sports competition movements during the intervention.

One limitation of this study is that the activity level was not assessed. According to Zhang et al. [30], CSA is reported to be affected by activity level; therefore, it is highly possible that differences in activity level affected the results of this study. Secondly, the results of this study are speculative because the mechanism of the arch support effect has not been investigated. Since the relationship between foot alignment and coarse toe flexor muscle strength has been shown in a previous study [31], it is necessary to conduct basic research on how individual muscle functions change with changes in muscle fiber length.

## Conclusions

It can be concluded that a 9-weeks arch support intervention may promote the development of ABH and FDL CSA in young soccer players. Additionally, with a 9-weeks arch support intervention, the CSA of the ABH and FDL in young soccer players with reduced MLA could develop 3–6% greater than that in the observation period.

## Data Availability

The datasets used and/or analysed during the current study are available from the corresponding author on reasonable request.

## Abbreviations

MLA: Medial longitudinal arch
CSA: Cross sectional area
ABH: Abductor halluces
FPI: Foot posture index-6
FHB: Flexor hallucis brevis
FDB: Flexor digitorum brevis
FHL: Flexor hallucis longus
PER: Peroneal muscle
TP: Tibialis posterior
PHVA: Peak height velocity age
